# Pharmacist-Led Interventions for Polypharmacy Management in Older Adults: A Systematic Review of Strategies and Outcomes in the United Kingdom and the Republic of Ireland

**DOI:** 10.3390/pharmacy13040109

**Published:** 2025-08-19

**Authors:** Fionnuala McGrory, Mohamed Hassan Elnaem

**Affiliations:** School of Pharmacy and Pharmaceutical Sciences, Ulster University, Coleraine BT52 1SA, UK

**Keywords:** polypharmacy, deprescribing, pharmacist, older adults, United Kingdom, Republic of Ireland

## Abstract

Polypharmacy in older adults increases risks of adverse drug reactions (ADRs), hospitalisations, and mortality. Pharmacist-led interventions using validated tools (e.g., STOPP/START, MAI, STOPPFrail) aim to optimise prescribing, yet their impact on clinical and economic outcomes in UK/Ireland health systems remains underexplored. This systematic review aimed to critically assess the impact of pharmacist-led deprescribing interventions on PIP, clinical outcomes, and costs in older adults (≥65 years) across the UK and Ireland. Following PRISMA 2020 guidelines, four databases (PubMed, Scopus, Web of Science, Cochrane Library) were searched for studies (2010–2024). Eligible studies included randomised trials, observational designs, and intervention studies in hospitals, care homes, community pharmacies, and intermediate care settings. Fourteen studies met the inclusion criteria. The risk of bias was assessed using CASP checklists. Narrative syntheses and heat maps summarised the findings. Twelve of fourteen studies reported positive outcomes: reductions in potentially inappropriate medications, ADRs, medication burdens, and falls/fall risks. Medication appropriateness improved significantly in 35% of studies using the MAI. STOPPFrail reduced PIMs in care homes, while the MAI enhanced complex hospital reviews. Community interventions improved adherence and reduced the use of fall risk drugs. No studies demonstrated a reduction in hospitalisations, mortality, or the length of stays. Economic analyses showed mixed cost-effectiveness. Key barriers included low uptake of pharmacist recommendations and short follow-up periods. Pharmacist-led interventions have significantly improved the prescribing quality and reduced medication-related risks, but they fail to impact hospitalisations or mortality due to implementation gaps. Context-specific tools and policy reforms—including expanded pharmacist roles and electronic decision support—are critical for sustainability. Future research should focus on long-term outcomes, cost-effectiveness, and multidisciplinary integration.

## 1. Introduction

Medication errors contribute significantly to avoidable hospitalisations in the UK and Ireland, costing the NHS over GBP 98 million annually [1]. Globally, the WHO’s Medication Without Harm initiative targets a 50% reduction in severe medication-related harm by focusing on polypharmacy, high-risk situations, and care transitions [2]. This review explicitly addresses polypharmacy—defined as the concurrent use of five or more medications [3]—which affects 23–29% of older adults in the UK [4]. It escalates risks of adverse drug reactions (ADRs), hospitalisations, and mortality. Critically, polypharmacy manifests as “appropriate” (necessary for complex comorbidities) or “problematic” (risks outweigh benefits), with the latter driving drug interactions, poor adherence, and reduced quality of life [5].

Potentially inappropriate prescribing (PIP)—encompassing inappropriate medications (PIMs) or omissions of essential treatments—is prevalent in 17–51% of older adults across the UK and Ireland [6,7,8]. While tools like STOPP (Screening Tool of Older Persons’ Prescriptions) and START (Screening Tool to Alert to Right Treatment) criteria [9], Beers Criteria [10], and the Medication Appropriateness Index (MAI) [11] have been used to mitigate PIP; their real-world impact, however, remains inconsistent. Pharmacist-led interventions show promise in optimising prescribing [12,13], yet systematic reviews report mixed outcomes on hospitalisations and mortality [14,15], underscoring gaps in evidence-based implementation.

The UK and Ireland share critical contextual factors that justify an independent investigation, such as demographic parallels: rapidly ageing populations with a comparable polypharmacy prevalence [4,7]. Additionally, both nations share a similar model of healthcare system: publicly funded models (NHS/HSE) facing comparable pressures from medication-related harm [1,16]. Moreover, they exhibit policy divergence: varied integration of pharmacists in care pathways (e.g., independent prescribing in the UK vs. limited authority in Ireland [17]). Finally, they exhibit variation in tool adoption strategies; for example, STOPP/START was developed in Ireland [9] and recommended by NICE [18] and is widely used regionally. However, its effectiveness relative to alternatives (e.g., DBI, MAI) is underexplored in this context.

There is a lack of recent syntheses evaluating which pharmacist-led strategies are most effective for managing polypharmacy within specific health systems. Regionally tailored evidence is crucial to address the suboptimal uptake of pharmacist recommendations [12,19], heterogeneity in outcomes across settings (e.g., hospitals vs. care homes), and the economic viability of interventions in resource-constrained systems [16,20]. This systematic review, therefore, aimed to critically assess the impact of pharmacist-led deprescribing interventions on PIP, clinical outcomes, and costs in older adults (≥65 years) across the UK and Ireland.

## 2. Methodology

This systematic review was conducted following the Preferred Reporting Items for Systematic Reviews and Meta-Analyses (PRISMA) 2020 guidelines to ensure methodological rigour, transparency, and reproducibility. The review protocol was registered with PROSPERO (Registration number: CRD420251002915) before the commencement of the study. No amendments were made to the original protocol during the conduct of this review.

### 2.1. Eligibility Criteria

Study Characteristics:

Population: Adults aged 65 years and older receiving healthcare services in the United Kingdom (England, Scotland, Wales, Northern Ireland) or the Republic of Ireland.

Intervention: Pharmacist-led interventions utilising validated deprescribing or medication screening tools, including the following: STOPP/START criteria, STOPPFrail, Drug Burden Index (DBI), anticholinergic burden (ACB) score, Medication Appropriateness Index (MAI), Beers Criteria, Priscus List, or other validated medication optimisation tools.

Comparator: No specific comparator was required due to the qualitative nature of this review, focusing on intervention effectiveness.

Outcomes: Primary outcomes included medication appropriateness, potentially inappropriate prescribing (PIP), polypharmacy burden, and adverse drug reactions (ADRs). Secondary outcomes included hospital admissions, readmissions, length of stay (LOS), mortality, falls, medication adherence, health-related quality of life (HRQOL), and comorbidity management.

Study Design: Randomised controlled trials (RCTs), observational studies (cohort, case-control), and intervention studies.

Setting: All healthcare settings, including hospitals, community pharmacies, care homes, intermediate care facilities, and general practice.

Report Characteristics:

Publication Period: Studies published from 1 January 2010 to 31 December 2024, to capture contemporary advances in polypharmacy management.

Language: English-language publications only.

Publication Status: Peer-reviewed original articles only.

Exclusion Criteria: Studies were deemed ineligible if (1) the participants were under 65 years of age, (2) the interventions were not pharmacist-led, (3) no validated screening tool was utilised, (4) the studies were conducted outside the UK or the Republic of Ireland, (5) no relevant clinical outcomes were measured or reported, or (6) the study design was inappropriate (case reports, narrative reviews, editorials).

### 2.2. Information Sources

A thorough search was conducted across four electronic databases: PubMed (MEDLINE), Scopus, Web of Science Core Collection, and the Cochrane Library. In addition, reference lists from the included studies and relevant systematic reviews were manually screened to identify any additional eligible studies. Forward and backwards citation searching was also performed using Google Scholar for all the included studies. No grey literature sources, clinical trial registries, or expert consultations were utilised, as the focus was solely on published, peer-reviewed evidence.

### 2.3. Search Strategy

Search strategies were developed using a combination of Medical Subject Headings (MeSH terms) for PubMed and the Cochrane Library, as well as free-text keywords for all databases. The search strategy was based on three main concept blocks: (1) polypharmacy and medication management, (2) pharmacist interventions, and (3) older adults and geographic location.

The search strategies were adapted for each database’s specific syntax and controlled vocabulary. Boolean operators (AND, OR) and truncation symbols were applied appropriately. Date limits (2010–2024) and English language restrictions were applied consistently across all the databases. Table S1 lists the MeSH terms and keywords.

### 2.4. Study Selection Process

Study selection was conducted in two phases by two independent reviewers (FM and ME). All the retrieved records were imported into RefWorks for duplicate removal and screening management. Both reviewers independently screened the titles and abstracts of all the retrieved records against the predetermined eligibility criteria. Disagreements were resolved through discussion, and a third reviewer was consulted if a consensus could not be reached. Full-text articles of potentially eligible studies were retrieved and independently assessed by both reviewers. Reasons for exclusion were documented using a standardised form. All disagreements between reviewers were resolved through discussion and reaching a consensus. A structured approach was used to document the rationale for inclusion or exclusion decisions. No translation services were required as only English-language publications were eligible.

### 2.5. Data Collection Process

Data extraction was performed using a standardised, piloted data extraction form developed specifically for this review. The form was tested on five studies and refined before full implementation. Discrepancies in data extraction were resolved through discussion between reviewers. When necessary, study authors were contacted via email to clarify missing or unclear data, with a maximum of two contact attempts made over four weeks. Extracted data were compiled in Microsoft Excel, with regular backup and version control procedures implemented.

### 2.6. Data Items

The primary outcome domains included the appropriateness of medication and the prevalence of potentially inappropriate prescribing, assessed using validated tools. Additionally, the burden of polypharmacy was measured by the number of medications and DBI (Drug Burden Index) scores, along with the incidence and severity of adverse drug reactions.

On the other hand, the secondary outcome domains comprised healthcare utilisation (including hospital admissions, readmissions, and emergency department visits), clinical outcomes (such as mortality rates, falls, and length of hospital stays), patient-reported outcomes (focusing on quality of life and medication adherence), and economic outcomes (encompassing cost-effectiveness and healthcare costs).

Other variables extracted, such as study characteristics, included population demographics, intervention details, study methodology, and characteristics of the healthcare setting. For managing missing data, when information was unavailable or unclear, assumptions were documented transparently.

### 2.7. Risk of Bias Assessment

The risk of bias was assessed using Critical Appraisal Skills Programme (CASP) checklists, with specific versions for different study designs. Key domains assessed included study validity and methodology appropriateness, research question clarity and focus, population recruitment and selection bias, data collection methods and outcome measurement, statistical analysis appropriateness, and results interpretation and clinical significance.

The risk of bias for each included study was assessed. Disagreements were resolved through open discussion and a consensus-based approach. Domain-specific assessments were reported to provide a nuanced evaluation of study quality. Tables S2 and S3 provide an overview of the RCT and cohort study CASP checklists, respectively.

### 2.8. Synthesis Methods

Studies were grouped for synthesis based on the intervention type, outcome similarity, and study design. Due to substantial clinical and methodological heterogeneity, a formal meta-analysis was not conducted. Instead, a structured narrative synthesis approach was employed. When studies reported multiple time points, the longest follow-up data were considered. Sensitivity analyses were not pre-specified due to the anticipated heterogeneity and descriptive nature of the synthesis.

### 2.9. Assessment of Reporting Biases and Certainty

Due to the limited number of studies per outcome and substantial heterogeneity, formal statistical tests for publication biases (such as funnel plots or Egger’s test) were not appropriate. The certainty of evidence was not formally assessed using the GRADE methodology due to the descriptive nature of this review and the substantial heterogeneity that precluded a meta-analysis. Instead, study quality was thoroughly assessed using CASP checklists, and the overall strength of evidence was discussed narratively in the context of study limitations, consistency of findings, and clinical relevance.

## 3. Results

**Figure 1:**
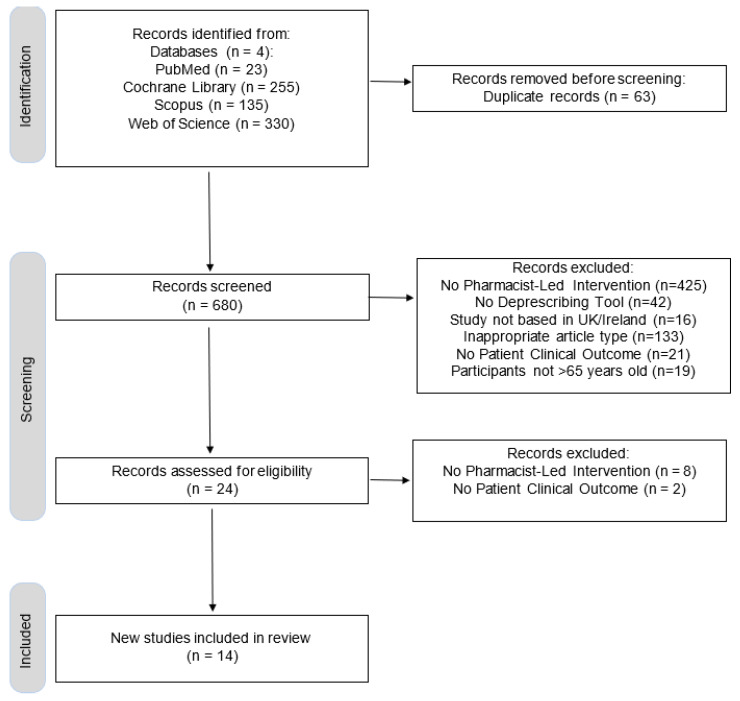
A PRISMA flow diagram illustrating the selection process.

This section presents the findings from the systematic review, including study characteristics, screening tools, clinical outcomes, quality analysis and economic impact. Of the 743 articles screened, 14 were included in this review for final analysis. Figure 1 highlights the screening process carried out in this systematic review.

### 3.1. Study Characteristics

A total of 14 studies published from 2010 onwards were included, which are summarised in Table 1. Concerning clinical settings, nine were conducted in hospitals, two in community pharmacies, two in care homes, and one in intermediate care. Different types of study designs were included in this review. Five studies were RCTs, five were observational studies, three were intervention studies, and one was an evaluation study. A wide variation in the number of participants in each study can be derived from Table 1. Approximately 50% of the trials included in this systematic review were based in the Republic of Ireland, while the other half were carried out in different regions of the UK. Study characteristics can be derived from Table 2.

### 3.2. Screening Tools

Various deprescribing tools were employed throughout eligible studies. STOPP/START was the most extensively used tool among these studies. However, other screening tools, including the DBI, MAI, Beers Criteria, Priscus List, and the ACB Calculator, were employed in combination. Other screening tools based on the STOPP/START framework were used, primarily targeting more specific populations, such as STOPPFrail, which is used for patients with frailty.

**Figure 2:**
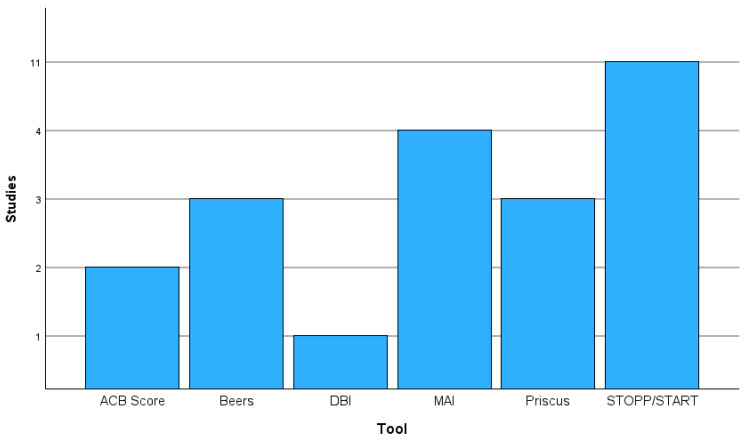
A bar chart displaying the frequency of specific screening tools in eligible studies. Some studies used multiple tools.

Figure 2 highlights the frequency with which each screening tool was utilised across eligible studies. Eleven studies utilised the STOPP/START framework to aid in the analysis of patient records. Four studies used the Medication Appropriateness Index. Three studies used the Beers Criteria and the Priscus List. Two studies utilised the ACB calculator, while only one study employed the Drug Burden Index.

### 3.3. Clinical Outcomes

The studies analysed a broad spectrum of clinical outcomes and evaluated the significance of pharmacist-led interventions in patient care and management. Twelve studies reported positive clinical outcomes, while one randomised controlled trial (RCT) did not yield favourable results. Additionally, a prospective cohort study, part of the intervention arm of an RCT, also failed to demonstrate positive clinical outcomes. Of the fourteen studies, two demonstrated a significant reduction in polypharmacy. Five studies observed notable increases in medication appropriateness following targeted interventions. Furthermore, three studies revealed improvements in reducing falls or fall risks among older adults through medication reviews. Two studies reported substantial reductions in the medication burden due to pharmacist-led interventions aimed at optimising medications for older adults. Additionally, one study highlighted a positive impact on medication adherence and quality of life scores. A detailed overview of these key clinical outcomes is summarised in Table 1, while Table 3 outlines the frequency of outcome reporting.

### 3.4. Quality Analysis

Overall, the majority of studies demonstrated a sound design and yielded reliable results, although notable limitations were observed in certain areas. The randomised controlled trials (RCTs) included in this review generally adhered to rigorous protocols and randomisation standards, resulting in varying outcomes, as outlined in Table 4. Despite these differences, the studies demonstrate a solid methodological design, practical implementation, and informative descriptive data. Table 5 offers an overview of the CASP checklist for cohort studies. Collectively, these studies present a compelling case for inclusion, providing robust observational evidence that supports the effectiveness of pharmacist-led medication optimisation utilising deprescribing tools among adults over 65. While methodological limitations are acknowledged, the consistency of the findings, the employment of validated assessment tools, and their relevance to clinical practice render them valuable contributions to the field.

### 3.5. Economic Impact Assessment

Five studies reported economic outcomes related to pharmacist-led interventions in older adults, as per Table 6. There were mixed findings across these studies regarding the overall cost-effectiveness of the intervention, with two studies not concluding that their implemented interventions were cost-effective.

## 4. Discussion

### 4.1. Key Findings and Context

Pharmacist-led interventions have shown a significant improvement in the prescribing quality within healthcare settings across the UK and Ireland. The use of structured tools—particularly the STOPP/START criteria, MAI, and STOPPFrail—has led to a reduction in PIMs [22,28], a decrease in the anticholinergic burden by 9.6–33% [22,26], and a reduction in hospital-acquired adverse drug reactions (ADRs) [19,24]. However, these improvements did not result in better overall clinical outcomes, as hospitalisations, mortality rates, and the length of stays remained unchanged in several studies. The effectiveness of these interventions was notably influenced by two key factors: (1) the contextual alignment of the tools used, where STOPPFrail’s frailty-specific criteria were more effective than generic instruments in care home settings [26]; and (2) implementation fidelity, as low adoption of recommendations undermined the potential benefits [12].

### 4.2. Tool-Specific Efficacy: Evidence for Contextual Adoption

This review supports the existing literature on the crucial role of pharmacists in managing patients with polypharmacy. Pharmacist-led interventions, utilising tools such as STOPP/START, STOPPFrail, and the MAI, have consistently demonstrated improvements in prescribing quality and clinical outcomes. STOPP criteria significantly reduced PIP compared to Beers Criteria and the Priscus List, which did not yield statistically significant results [28]. Reducing PIMs and the anticholinergic burden in nursing homes was achieved through the STOPPFrail program, demonstrating effectiveness in addressing polypharmacy [23,26].

The MAI tool is the second most commonly used for reducing medication appropriateness, but its complexity may limit its practicality under staffing pressures. Meanwhile, STOPP/START is the most sensitive tool compared to Beers Criteria and the MAI, though studies have shown poor adherence to its recommendations. To improve outcomes, integrating STOPP/START into clinical practice with electronic decision support systems is crucial. Additionally, training multidisciplinary teams on these tools, particularly adapted versions like STOPPFrail, can enhance outcomes for frail older adults by promoting a more patient-centred approach. The effectiveness of particular tools can be illustrated through a comparative analysis of outcomes. STOPPFrail has successfully achieved a sustained reduction in PIM within care homes. This outcome underscores STOPPFrail’s emphasis on addressing frailty-specific risks, such as CNS drug toxicity, which is particularly important considering the high baseline prevalence of PIM in nursing homes.

### 4.3. Outcomes in Various Clinical Settings

Seven out of nine hospital-based studies reported positive outcomes, including reductions in medication appropriateness, hospital-acquired adverse drug reactions (ADRs), fall risk medications, polypharmacy, and comorbidities. One study in Wales reported a decrease in comorbidities after using the STOPP/START criteria in psychiatry. However, polypharmacy remained unaffected, underscoring the need for tailored interventions in various clinical settings [21]. Programs like Pharm2Pharm in the U.S. suggest that integrating pharmacists into care transitions can reduce hospitalisations [32]. Future research should aim to incorporate pharmacists within multidisciplinary teams to improve patient-centred outcomes.

Both studies in community pharmacies showed positive outcomes, presented as an overall mix of reported improvements in medication adherence, quality of life, and fewer falls, as well as reductions in polypharmacy, fall risks, and the anticholinergic cognitive burden [22,31]. These results underscore the value of community-based services. However, no studies have focused on community pharmacies in the Republic of Ireland, where the roles of pharmacists and independent prescribers are being debated. This suggests that the potential for pharmacist-led interventions in Ireland is underexplored. Additionally, polypharmacy is a common issue, indicating the need for regular interventions across all healthcare settings.

One study in this review examined an intermediate care setting and found that lowering MAI scores did not lead to fewer unplanned hospital readmissions [25]. This suggests that such interventions may not improve long-term clinical outcomes or reduce the burden on the healthcare system. In contrast, a study in Northern Ireland evaluated the impact of a consultant pharmacist, which showed safer, more streamlined, and cost-effective prescribing [33]. This highlights the crucial role of pharmacist-led interventions in improving patient outcomes across various clinical settings.

In care homes, the STOPPFrail tool has shown significant utility in supporting rational deprescribing decisions [26]. In contrast, utilising the STOPP criteria in a randomised controlled trial improved medication appropriateness but did not reduce emergency visits, falls, or mortality, and faced challenges with participant retention and baseline medication discrepancies [16]. These findings highlight the prevalence of PIMs in care homes and the benefits of pharmacist-led interventions, utilising tools such as STOPPFrail. Future research should assess long-term outcomes, such as hospital admissions and quality of life, with a focus on sustained deprescribing and addressing the limitations of prior studies. It should also explore the systematic implementation of these tools in routine care.

Hospital-based studies within this review revealed mixed outcomes, highlighting both the potential benefits and limitations of deprescribing tools in acute care. These interventions are vital for older adults with complex polypharmacy, who are at an increased risk of adverse drug events during transitions of care. Overall, outcomes such as inappropriate prescribing and medication adherence showed more positive outcomes. Research by Sallevelt et al. indicated that the use of STOPP/START in hospitals did not result in a significant reduction in hospital readmissions [29]. One study found no benefits associated with implementing the STOPP/START criteria, which was likely hindered by the low uptake of recommendations [12]. Future research should include larger and more diverse populations, prioritise comprehensive evaluations, and implement pharmacist-led screening tools to enhance findings across various clinical outcomes. Additionally, it is important to analyse the rationale behind pharmacotherapy recommendations to improve outcome reliability.

### 4.4. Policy Implications: Evidence-Backed Recommendations

Collective evidence underpins the valuable role of pharmacists in identifying PIMs, enhancing medication appropriateness, and reducing polypharmacy in older adults. Community pharmacy studies have shown positive improvements in adherence, reduced fall risks, and support for deprescribing. This reinforces the potential of community pharmacists to contribute more effectively to this context, given that the legislative reform has been made. In intermediate and secondary care, pharmacist-led interventions often improved process outcomes but failed to achieve long-term clinical outcome endpoints, highlighting the need for widespread, systematic changes in implementing deprescribing within these clinical settings.

This systematic review offers several recommendations that could significantly impact policy. Firstly, it is crucial to prioritise the STOPPFrail framework in care homes, where frailty-specific deprescribing has consistently resulted in reductions in PIMs, thereby addressing a vital unmet need. Secondly, for complex hospital reviews within a clinical context, a combination of tools is strongly recommended. This approach integrates the qualitative methods of the MAI with adoption-efficient tools to more effectively minimise inappropriate prescriptions. Thirdly, expanding the roles of community pharmacists, especially in Ireland, is advised, as their services have demonstrated improvements in medication adherence and quality of life. Finally, beyond current evidence, it is essential to implement electronic decision support systems to enhance the uptake of recommendations and to consider providing financial incentives for high-value interventions, which can help offset short-term cost increases.

### 4.5. Limitations and Future Research Priorities

A significant strength of this systematic review lies in its wide range of study designs and healthcare settings, enabling a thorough analysis of pharmacist-led interventions across secondary, intermediate, residential care, and community pharmacy environments. However, this review has several limitations. First, several studies were hindered by small sample sizes, which may have restricted their statistical power and generalisability. Second, the limited uptake of pharmacist recommendations signifies a potential disconnect within the multidisciplinary team and ultimately raises questions about the reliability of the data generated. Third, the prevalence of single-point medication reviews and short follow-up periods may account for the lack of significant findings regarding hospital admissions and mortality. Fourth, there is a notable absence of direct comparisons between tools, with only three studies utilising multiple instruments, alongside a variety of economic methodologies. Finally, this review did not capture any eligible studies based on general practice, a role that may be expanded further in the future, and additional studies are required to determine outcomes in this setting.

Future research should prioritise conducting randomised controlled trials that directly compare screening tools in care homes. It is also crucial to assess the cost-effectiveness of pharmacist prescribing in Irish community pharmacies, where legislative barriers persist. Additionally, extending follow-up periods beyond 12 months would provide valuable insights into the long-term impacts on mortality and hospitalisations. Finally, studies should explore the barriers and facilitators to accepting pharmacist recommendations, particularly in the Republic of Ireland, where the pharmacist’s role has not been expanded to include that of an independent prescriber, as in the UK.

## 5. Conclusions

This systematic review synthesises evidence from the UK and the Republic of Ireland demonstrating that pharmacist-led interventions using validated tools significantly improve medication appropriateness, reduce PIP, and lower ADRs and medication burdens in older adults. However, these benefits did not extend to reductions in hospitalisations, mortality, or the length of stays, likely due to limitations such as low implementation fidelity (e.g., poor uptake of pharmacist recommendations), short follow-up periods, and insufficient sample sizes. The efficacy of interventions varied across settings: STOPPFrail excelled in care homes for deprescribing related to frailty, the MAI enhanced complex hospital reviews, and community-based services improved medication adherence and fall risk management.

To achieve sustainable impacts, future initiatives must incorporate context-specific tools, such as STOPPFrail in care homes, into routine practice through electronic decision support and multidisciplinary collaboration. It is essential to address implementation barriers by advocating for policy reforms that include expanding the roles of pharmacists in Ireland and providing financial incentives for high-value interventions. Additionally, prioritising research on long-term outcomes and making direct comparisons of tools and cost-effectiveness in community settings are crucial. Ultimately, the integration of these evidence-based strategies into healthcare policy and clinical practice is vital for mitigating the risks associated with polypharmacy and advancing patient-centred care for ageing populations.

## Figures and Tables

**Table 1:** A summary of the key characteristics of the included studies (*n* = 14).

Author & Year	Setting	Region	Study Design	Sample Size	Screening Tool	Clinical Outcome	Conclusion
Aziz et al., 2018 [21]	Hospital	Wales	Observational Audit	*n* = 86	STOPP/START	Comorbidities, Total Prescriptions	Significant reductions occurred in comorbidities and the number of prescriptions. No clinically significant differences were observed for the reduction of polypharmacy or the number of patients in a specialist dementia ward.
Crawford et al., 2024 [22]	Community	Northern Ireland	Intervention Study	*n* = 92	ACBCalc^®^, MAI	Medication Appropriateness, Falls	Post-intervention analysis revealed a reduction in polypharmacy and an improvement in the ACB score. There was an improvement in medication appropriateness, and a clinically significant reduction in exposure to fall-related medications occurred.
Curtin et al., 2020 [23]	Hospital	Ireland	RCT	*n* = 130	STOPPFrail	PIMs, Falls, Hospitalisations, Fractures, Mortality and QOL	This trial significantly reduced polypharmacy; however, there were no differences in terms of other health outcomes, such as hospitalisations, falls, fractures, QOL, and mortality.
Dalton et al., 2019 [24]	Hospital	Ireland	RCT	*n* = 285	STOPP/ START, Beers Criteria, and Priscus List	ADRs, PPOs, Hospital LOS, Mortality Rate	Pharmacist-led interventions resulted in a significant reduction in ADRs and significant implementation of the START criteria. There were no significant differences in mortality rates or hospital LOS.
Desborough et al., 2020 [16]	Care Home	England	RCT	*n* = 826	STOPP	Falls, Emergency Visits, Mortality	There were significant reductions in PIMs after 12 months; however, the difference was borderline significant after 6 months. There was no significant reduction in falls, emergency visits, or survival.
Doherty et al., 2022 [25]	Intermediate Care	Northern Ireland	Observational Study	*n* = 532	MAI	Medication Appropriateness, Hospital Readmissions	There were significant reductions in the MAI from admission to discharge. There were no significant differences in hospital readmissions; however, those who received educational intervention were less likely to be readmitted to acute care.
Hurley et al., 2024 [26]	Care Home	Ireland	Intervention Study	*n* = 99	STOPPFrail, DBI Score, and ACB Score	Medication Burden, MAI, Falls, Hospitalisation, Emergency Visits, HRQOL, and Mortality Rates	This study found a significant reduction in the medication burden. There were no significant falls, hospitalisations, or mortality increases, highlighting the safe implementation of deprescribing. However, there were no significant improvements in falls, emergency visits or QOL. DBI and ACB scores significantly decreased post-review, suggesting reduced medicine-related sedation and frailty and increased medication appropriateness.
Marvin et al., 2017 [27]	Hospital	England	Observational Study	*n* = 100	STOPP and STOPIT	Fall Risk	Reduction of fall risk medications.
O’Mahoney et al., 2020 [12]	Hospital	Ireland & Scotland	RCT	*n* = 1537	STOPP/START	ADRs, All-Cause Mortality Rates, Hospital Readmission Rates, HRQOL	The intervention did not significantly improve clinical outcomes, possibly due to a 15% adherence to recommendations. No impact was found regarding the reduction of ADRs, mortality, readmission, or QOL.
O’Sullivan et al., 2014 [28]	Hospital	Ireland	Intervention Study	*n* = 361	STOPP/ START, Beers Criteria, and Priscus List, MAI	Medication Appropriateness	Statistically significant improvement in MAI scores after the intervention, along with a significant reduction in PIP by STOPP criteria. Whilst Beers and Priscus showed slight improvements in PIP, they were not statistically significant.
O’Sullivan et al., 2016 [19]	Hospital	Ireland	RCT	*n* = 737	STOPP/ START, Beers Criteria, and Priscus List	ADRs, Hospital LOS, All-Cause Mortality	Significant reduction in hospital-acquired ADRs. No effect on hospital LOS or all-cause mortality.
Sallevelt et al., 2022 [29]	Hospital	Ireland (Multicentre)	Observational Study	*n* = 963	STOPP/START	Drug-Related Admissions	STOPP/START medication reviews did not significantly reduce the occurrence of drug-related hospital admissions.
Tallon et al., 2016 [30]	Hospital	Ireland	Observational study	*n* = 108	MAI	Medication Appropriateness	The application of the MAI significantly improved medication appropriateness and reduced the number of inappropriate prescriptions at discharge.
Twigg et al., 2015 [31]	Community	England	Evaluation Study	*n* = 620	STOPP/START	Fall Risk, Pain Management, Medication Adherence, HRQOL	Significant reduction in falls, increase in medication adherence and QOL (EQ-5D-5L scores). No significant changes were observed in pain scores.

ACB: anticholinergic burden; ADRs: adverse drug reactions; DBI: Drug Burden Index; HRQOL: health-related quality of life; LOS: length of stay; MAI: Medication Appropriateness Index; PIMs: Potentially Inappropriate Medicines; PIP: potentially inappropriate prescribing; PPOs: Potential Prescribing Omissions; RCTs: randomised controlled trial.

**Table 2:** A summary of the clinical setting, study design and location of the included studies.

Clinical Setting	Studies, N (%)
Hospital	9 (65%)
Care Home	2 (14%)
Community	2 (14%)
Intermediate Care	1 (7%)
**Study Design**	
RCT	5 (36%)
Observational Study	5 (36%)
Intervention Study	3 (21%)
Evaluation Study	1 (7%)
**Study Location**	
England	3 (20%)
Scotland	1 (7%)
Wales	1 (7%)
Northern Ireland	2 (13%)
Republic of Ireland	8 (53%)

**Table 3:** Number of studies with positive and adverse clinical outcomes assessed and frequency of outcomes not assessed.

Clinical Outcome	Positive Outcome Observed, N (%)	No Positive Outcome, N (%)	Outcome Assessed, N (%)	Outcome Not Assessed, N (%)
Improved Medication Appropriateness	5 (35%)	0 (0%)	5 (35%)	9 (65%)
Reduction of Polypharmacy	2 (14%)	2 (14%)	4 (28%)	10 (72%)
Reduction of Falls or Fall Risk Medicine	3 (21%)	3 (21%)	6 (43%)	8 (57%)
Reduction of Inappropriate Prescribing	1 (7%)	0 (0%)	1 (7%)	13 (93%)
Reduction of Adverse Drug Reactions	2 (14%)	2 (14%)	4 (28)	10 (72%)
Reduction of Medication Burden	2 (14%)	0 (0%)	2 (14%)	12 (86%)
Reduction in Comorbidities	1 (7%)	0 (0%)	1 (7%)	13 (93%)
Improved Medication Adherence	1 (7%)	0 (0%)	1 (7%)	13 (93%)
Improved Quality of Life	1 (7%)	3 (21%)	4 (28%)	10 (72%)
Reduction in Hospitalisation	0 (0%)	6 (43%)	6 (43%)	8 (57%)
Improved Mortality Rates	0 (0%)	6 (43%)	6 (43%)	8 (57%)
Reduction in Hospital LOS	0 (0%)	3 (21%)	3 (21%)	11 (79%)

**Table 4:** A summary of the quality assessment of randomised controlled trials using the CASP RCT checklist.

Selection/Topic	Item	Curtin et al. [23]	Dalton et al. [24]	Desborough et al. [16]	O’Mahoney et al. [12]	O’Sullivan et al. [19]
Research Question	Q1	Yes	Yes	Yes	Yes	Yes
Randomisation	Q2	Yes	Yes	Yes	Yes	Yes
Patients Accounted for at Conclusion	Q3	Yes	Yes	No	Yes	Yes
Blinding of Participants	Q4a	No	No	No	No	No
Blinding of Investigators	Q4b	No	No	No	No	No
Blinding of Assessors	Q4c	Yes	No	Yes	Yes	Yes
Similarity of Study Groups	Q5	Yes	Yes	No	Yes	Yes
Equal Care within Study Groups	Q6	Yes	Yes	Yes	Yes	Yes
Comprehensive Reporting of Effects	Q7	Yes	Yes	Yes	Yes	Yes
Reporting of Precision of Effects	Q8	Yes	Yes	Yes	Yes	Yes
Benefits Outweigh the Risks	Q9	Yes	Yes	No	No	Yes
Applicability to Context/Locality	Q10	Yes	Yes	Yes	Can’t Tell	Yes
Value of Intervention Versus Existing Interventions	Q11	Yes	Yes	No	No	Yes

**Table 5:** A summary of the study quality assessment of cohort studies using the CASP cohort checklist.

Selection/Topic	Aziz et al. [21]	Crawford et al. [22]	Doherty et al. [25]	Hurley et al. [26]	Marvin et al. [27]	O’Sullivan et al. [28]	Sallevelt et al. [29]	Tallon et al. [30]	Twigg et al. [31]
Clear Focused Issue	Yes	Yes	Yes	Yes	Yes	Yes	Yes	Yes	Yes
Acceptable Recruitment	Yes	Yes	Yes	Yes	Yes	Yes	Yes	Yes	Yes
Accurate Exposure Measurement	Yes	Yes	Yes	Yes	Yes	Yes	Yes	Yes	Yes
Accurate Outcome Measurement	Yes	Yes	Yes	Yes	Yes	Yes	Yes	Yes	Yes
Identification of Confounding Factors	Yes	Yes	Yes	No	Cannot Tell	Yes	Yes	Cannot Tell	No
Account of Confounding Factors in Study Design	Yes	Yes	Yes	No	Cannot Tell	Yes	Yes	No	No
Subject Follow-Up Complete	Yes	Yes	Yes	Yes	Yes	Yes	Yes	Yes	No
Appropriate Follow- Up Time	Yes	Yes	Yes	Cannot Tell	No	Yes	Yes	Yes	No
Results	Yes	Yes	Yes	Yes	Yes	Yes	Yes	Yes	Yes
Precision of Results	Yes	Yes	Yes	Yes	Yes	Yes	Yes	Yes	Yes
Belief of Results	Yes	Yes	Yes	Yes	Yes	Yes	Yes	Yes	Yes
Applicability to Local Population	Yes	Yes	Yes	Yes	Yes	Yes	Yes	Yes	Yes
Results Fit Available Evidence	Yes	Yes	Yes	Yes	Yes	Yes	Yes	Yes	Yes
Implications for Practice	Yes	Yes	Yes	Yes	Yes	Yes	Yes	Yes	Yes

**Table 6:** An overview of economic implications of pharmacist-led interventions in adults over 65.

Study/(Tool)	Economic Outcome Assessment	Conclusion
Crawford et al. [22] (ACBCalc, MAI)	Annual cost avoidance and drug cost savings of GBP 40–80 K.	Cost-effective
Curtin et al. [23] (STOPPFrail)	Monthly medication cost Reduction of USD 60 ± USD 25 (*p* = 0.02) after 3 months.	Cost-effective
Desborough et al. [16] (STOPP)	Intervention mean cost per resident was GBP 375 higher than control.	Not cost-effective
Hurley et al. [26] (STOPPFrail, DBI, ACBCalc)	No reduction in mean monthly costs after a 6- month follow-up.	Not cost-effective
Twigg et al. [31] (STOPP/START)	Cost per quality-adjusted life year estimates from GBP 11–32 K.	Potential cost-effectiveness

## Data Availability

The raw data supporting the conclusions of this article will be made available by the authors on request.

## Supplementary Materials

The following supporting information can be downloaded at https://www.mdpi.com/article/10.3390/pharmacy13040109/s1. Table S1: MeSH Terms and Keywords; Table S2: Randomised Controlled Trial CASP Checklist; Table S3: Cohort Study CASP Checklist.
