# Supplementary material for: Pharmacist-Led Interventions for Polypharmacy Management in Older Adults: A Systematic Review of Strategies and Outcomes in the United Kingdom and the Republic of Ireland
Source: Pharmacy (Basel). 2025 Aug 19;13(4):109. doi: 10.3390/pharmacy13040109 (PMC12389713; doi:10.3390/pharmacy13040109)
Supplement: Supplementary file 1 [file pharmacy-13-00109-s001.zip › pharmacy-3706605-supplementary.pdf]

Supplementary Materials

Table S1: MeSH Terms and Keywords

PubMed

**Search Concept**	**Search Terms** (MeSH terms are bold)	**Total Results**
Polypharmacy	**polypharmacy** OR 'multiple medic*' OR 'inappropriate prescribing'	19,595
Pharmacist-led Intervention	**pharmaceutical service** OR 'medic* therap* management' OR pharmacist-led intervention* OR 'medic* review' OR '**deprescribing**' OR 'medic* optimization*'	9,220
Clinical Outcomes	hospital?ation* OR 'emergency department visit*' OR **'adverse drug reaction**' OR 'clinical outcome*' OR 'health complication*' OR 'healthcare event*' OR 'fall*'	43,833
Adults over 65 Years	**aged** OR 'older adults' OR 'elderly' OR 'geriatric patients'	6,443,308
United Kingdom and Republic of Ireland	**United Kingdom** OR 'UK' OR 'England' OR 'Scotland' OR 'Wales' OR 'Northern Ireland' OR '**Ireland**' OR 'Republic of Ireland'	69,427
**Total Results with 'AND'**		71
**Total Results with 'AND' plus Limits Applied**		23

Cochrane Library

**Search Concept**	**Search Terms**<br>(MeSH terms are bold.)	**Total Results**
Polypharmacy	**polypharmacy** OR 'multiple medic*' OR 'inappropriate prescribing'	25,534
Pharmacist-led Intervention	**pharmaceutical services** OR 'medic* therap* management' OR pharmacist-led intervention* OR 'medic* review' OR 'deprescribing' OR 'medic* optimization*'	757,712
Clinical Outcomes	**hospitalization** OR 'emergency department visit*' OR 'adverse drug reaction' OR 'clinical outcome*' OR 'health complication*' OR 'healthcare event*' OR 'fall*'	663,132
Adults over 65 Years	**aged** OR 'older adults' OR 'elderly' OR 'geriatric patients'	708,348
United Kingdom and Republic of Ireland	**United Kingdom** OR 'UK' OR 'England' OR 'Scotland' OR 'Wales' OR 'Northern Ireland' OR '**Ireland**' OR 'Republic of Ireland'	148,521
**Total Results with 'AND'**		277
**Total Results with ‘AND’ plus Limits Applied**		255

Scopus

Search Concept	Search Terms	Total Results
Polypharmacy	polypharmacy OR 'multiple medic*' OR 'inappropriate prescribing'	2,939
Pharmacist-led Intervention	pharmaceutical service* OR 'medic* therap* management' OR pharmacist-led intervention* OR 'medic* review' OR 'deprescribing' OR 'medic* optimization*'	33,549
Clinical Outcomes	hospitalization* OR 'emergency department visit*' OR 'adverse drug reaction*' OR 'clinical outcome*' OR 'health complication*' OR 'healthcare event*' OR 'fall*'	2,125,816
Adults over 65 Years	aged OR 'older adults' OR 'elderly' OR 'geriatric patients'	5,410,487
United Kingdom and Republic of Ireland	United Kingdom OR 'UK' OR 'England' OR 'Scotland' OR 'Wales' OR 'Northern Ireland' OR 'Ireland' OR 'Republic of Ireland'	13,128
**Total Results with 'AND'**		258
**Total Results with ‘AND’ plus Limits Applied**		135

Web of Science

Search Concept	Search Terms	Total Results
Polypharmacy	polypharmacy OR 'multiple medic*' OR 'inappropriate prescribing'	1,127,325
Pharmacist-led Intervention	pharmaceutical service* OR 'medic* therap* management' OR pharmacist-led intervention* OR 'medic* review' OR 'deprescribing' OR 'medic* optimization*'	2,660,824
Clinical Outcomes	hospital?ation* OR 'emergency department visit*' OR 'adverse drug reaction*' OR 'clinical outcome*' OR 'health complication*' OR 'healthcare event*' OR 'fall*'	4,375,205
Adults over 65 Years	aged OR 'older adults' OR 'elderly' OR 'geriatric patients'	12,740,565
United Kingdom and Republic of Ireland	United Kingdom OR 'UK' OR 'England' OR 'Scotland' OR 'Wales' OR 'Northern Ireland' OR 'Ireland' OR 'Republic of Ireland'	1,763,956
**Total Results with 'AND'**		1534
**Total Results with 'AND' plus Limits Applied**		330

Table S2: Randomised Controlled Trial CASP Checklist

Section A Is the basic study design valid for a randomised controlled trial?
Q1. Did the study address a clearly formulated research question? Yes/No/Can't Tell
Q2. Was the assignment of participants to interventions randomised? Yes/No/Can't Tell
Q3. Were all participants who entered the study accounted for at its conclusion? Yes/No/Can't Tell
Section B Was the study methodologically sound?
Q4a. Were the participants 'blind' to intervention they were given? Yes/No/Can't Tell
Q4b. Were the investigators 'blind' to the intervention they were giving to participants? Yes/No/Can't Tell
Q4c. Were the people assessing/analysing outcome/s 'blinded'? Yes/No/Can't Tell
Q5. Were the study groups similar at the start of the randomised controlled trial? Yes/No/Can't Tell
Q6. Apart from the experimental intervention, did each study group receive the same level of care (that is, were they treated equally)? Yes/No/Can't Tell
Section C: What are the results?
Q7. Were the effects of intervention reported comprehensively? Yes/No/Can't Tell
Q8. Was the precision of the estimate of the intervention or treatment effect reported? Yes/No/Can't Tell
Q9. Do the benefits of the experimental intervention outweigh the harms and costs? Yes/No/Can't Tell
Section D: Will the results help locally?
Q10. Can the results be applied to your local population/in your context? Yes/No/Can't Tell
Q11. Would the experimental intervention provide greater value to the people in your care than any of the existing interventions? Yes/No/Can't Tell

Table S3: Cohort Study CASP Checklist

Section A: Are the results valid?
Q1. Did the study address a clearly focused issue? Yes/No/Can't Tell
Q2. Was the cohort recruited in an acceptable way? Yes/No/Can't Tell
Q3. Was the exposure accurately measured to minimise bias? Yes/No/Can't Tell
Q4. Was the outcome accurately measured to minimise bias? Yes/No/Can't Tell
Q5a. Have the authors identified all important confounding factors? Yes/No/Can't Tell
Q5b. Have they taken account of the confounding factors in the design and/or analysis? Yes/No/Can't Tell
Q6a. Was the follow-up of subjects complete enough? Yes/No/Can't Tell
Q6b. Was the follow-up of subjects long enough? Yes/No/Can't Tell
Section B: What are the results?
Q7. What are the results of this study? Yes/No/Can't Tell
Q8. How precise are the results? Yes/No/Can't Tell
Q9. Do you believe the results? Yes/No/Can't Tell
Section C: Will the results help locally?
Q10. Can the results be applied to the local population? Yes/No/Can't Tell
Q.11 Do the results of this study fit with other available evidence? Yes/No/Can't Tell
Q12. What are the implications of this study for practice? Yes/No/Can't Tell
